# The Efficacy and Safety of Nicorandil for Periprocedural Myocardial Injury in Patients Undergoing PCI: A Meta-Analysis

**DOI:** 10.1155/2020/3293587

**Published:** 2020-11-06

**Authors:** Yuanxi Lu, Wenbiao Hu, Qinghua Song, Qiwu Wang

**Affiliations:** Department of Cardiology, The Second People's Hospital of Nanning, The Third Affiliated Hospital of Guangxi Medical University, Nanning, Guangxi, China

## Abstract

**Purpose:**

To evaluate the efficacy and safety of nicorandil for periprocedural myocardial injury in patients undergoing PCI through meta-analysis of randomized controlled trials.

**Methods:**

We analyzed the clinical data of patients including the incidence of periprocedural myocardial injury (PMI) and major adverse cardiovascular events (MACE) from selected articles. RCTs were retrieved from medical literature databases. RR and 95% confidence intervals (CI) were calculated to compare the endpoints.

**Results:**

In total, 15 articles (16 trial comparisons) were retrieved which contained 2221 patients. In general, 1130 patients (50.9%) were randomized to the experimental group, whereas 1091 patients (49.1%) were randomized to the control group. The result showed that nicorandil significantly reduced the incidence of PMI and MACE after PCI compared to the control group.

**Conclusions:**

Overall, early use of nicorandil in patients undergoing percutaneous coronary intervention (PCI) was associated with a significant reduction of PMI and MACE.

## 1. Introduction

Percutaneous coronary intervention (PCI) is a nonsurgical procedure used to treat narrowing of the coronary arteries of the heart found in coronary artery disease [1]. In patients with a restricted or blocked coronary artery, PCI may be the best option to reestablish blood flow as well as prevent angina (chest pain), myocardial infarctions (heart attacks), and death [2]. Thus, PCI is widely practiced in clinics.

Percutaneous coronary intervention (PCI) is associated with a small but significant incidence of serious procedural complications such as death, stroke, life-threatening bleeding, or large myocardial infarction (MI) [3]. Periprocedural myocardial injury, which can range from a low-level elevation of cardiac biomarkers to a large MI, is the most common complication and causes high mortality and prolonged hospital stays. The main causes of perioperative myocardial injury after PCI are distal embolization, side-branch occlusion, coronary dissection, and disruption of collateral flow [4].

Periprocedural myocardial injury includes angiographical slow coronary flow, microvascular embolization, and elevated levels of cardiac enzyme, such as creatine kinase and troponin-T and -I. Myocardial reperfusion injury at the beginning of myocardial reperfusion, which causes tissue damage and cardiac dysfunction, may also occur in cases of acute coronary syndrome [5].

Specific biomarkers are used for quantitative diagnosis of irreversible myocardial injury, and the release of these biomarkers is associated with increased risk of death and myocardial infarction (MI) [6]. A previous report showed that serum creatine kinase MB fraction (CK-MB) is elevated above the upper limit of normal (ULN) in 10 to 38 percent of patients after an uncomplicated percutaneous coronary intervention (PCI), and elevations more than three times the ULN are considered to represent an infarction large enough to be associated with short-term complications in 7 to 18 percent of patients [7]. Another report also indicated that a 5-fold postprocedural elevation of cardiac troponin-T above normal levels is an independent predictor of a composite of death, MI, and revascularization at 1 year (hazard ratio, 2.39; 95% confidence interval, 1.09–5.26) [8].

Because PMI also occurs in a significant proportion of patients with successful surgery, and the patients with myocardial injury are associated with a wide range of myocardial infarction, the long-term prognosis is also worse than those without myocardial injury [9].

Pharmacologic interventions, such as statins and glycoprotein IIb/IIIa inhibitors, which have anti-inflammatory and antithrombotic effects, respectively, are usually used before PCI and have been shown to reduce the incidence and degree of myocardial necrosis [10]. However, articles have shown that cardiac troponin levels are still elevated in 29% of patients after PCI, and the incidence of PCI-related myocardial infarction is still as high as 15% [11].

Nitrate may have a cardioprotective effect and is an independent factor for the outcomes of patients with PMI, and nicorandil has a potential dose-dependent protective effect for cardiac ischemia [12]. Nicorandil is an antianginal medication that has the dual properties of a nitrate and ATP-sensitive K^+^ channel agonist. In humans, the nitrate action of nicorandil dilates the large coronary arteries at low plasma concentrations. At high plasma concentration, nicorandil decreased coronary resistance, which was related to the increase of (KATP) opening of ATP-sensitive potassium channel [13]. The pharmacological treatment of ATP-sensitive potassium channel openers has a cardioprotective effect similar to that of ischemic preconditioning (IPC: brief episodes of cardiac ischemia and reperfusion before a subsequent prolonged ischemia), and the effect of ischemic preconditioning helps to avoid PMI [14]. Therefore, nicorandil has important therapeutic significance for myocardial injury after PCI.

However, the conclusion of whether nicorandil has myocardial protective effect is not consistent in clinic. Some studies showed that oral administration of nicorandil reduced the incidence of major cardiovascular events in patients with angina pectoris [15]. However, in other trials, intravenous nicorandil did not reduce the incidence of PMI or the slow-flow phenomenon following elective PCI, and certain studies refuted the beneficial effect of nicorandil against ischemia and reperfusion injury [8].

Thus, it is still uncertain whether nicorandil can effectively prevent PMI after PCI; we have carried on the systematic meta-analysis to this.

## 2. Methods

### 2.1. Search Strategy

Based on the Preferred Reporting Items for Systematic Reviews and Meta-Analyses (PRISMA) guidelines [16], two authors, respectively, searched the literature on evaluating the safety and efficacy of nicorandil in percutaneous coronary intervention (PCI). We comprehensively researched for RCTs in the data platforms including Embase, PubMed, and Cochrane Library without limitations on language or date of publication from inception to January 27, 2020. We used “nicorandil” and “percutaneous coronary intervention” as the key words. Additional relevant literature was obtained from other reviews and meta-analyses. The retrieved studies were double-reviewed by two authors. When there were disputes, a third author was asked to consult. In this meta-analysis, we obtained patient information from already published reports, and hence, ethical approval or patient consent was waived.

### 2.2. Criteria for Exclusion and Inclusion of Studies

Inclusion criteria were as follows: (1) studies that provided sufficient data for analysis; (2) studies that evaluated the safety and efficacy of nicorandil in percutaneous coronary intervention (PCI); (3) patients treated by PCI; (4) studies where at least one group was treated with nicorandil; (5) the use of nicorandil being not limited in dose and usage; (7) studies published in English; (8) the participants being Asian; and (9) randomized controlled trials (RCTs).

Exclusion criteria were as follows: (1) animal experiments; (2) case reports, nonclinical trials, or series; (3) nonrandomized trials or semirandomized controlled trials; and (4) studies with incomplete or incorrect information, or those with information that cannot be accessed.

### 2.3. Endpoints

The primary effective endpoint was perioperative myocardial injury (PMI).The safety endpoint was major adverse cardiovascular events (MACE).

### 2.4. Data Extraction

The studies included were scrutinized by two investigators independently to extract data. These investigators retrieved data regarding the primary endpoints and these data were verified by a third author. The following information was extracted: primary information: endpoints measured in each study, follow-up time, intervention, smoking, diabetes, BMI, average age, sex ratio, sample size, country of patients, year of publication, and first author's name. For missing data or the need for clarification, we contacted the first author to access such data. Any discrepancies were addressed by consensus or by involvement of a third author.

### 2.5. Assessment of the Risk of Bias

The Cochrane Risk of Bias criteria [17] were used to assess the methodological quality of the articles studies which was performed by two authors working independently. The quality level of the studies was categorised as high risk, low risk, or some concerns. The bias of each trial was assessed by 5 items as follows: bias arising from the randomization process, bias due to deviations from intended interventions, bias due to missing outcome data, bias in measurement of the outcome, and bias in selection of the reported result.

### 2.6. Data Analysis

Individual data were pooled and analyzed using the Stata (version 12.0, Stata Corp, College Station, Texas). Pooled data are presented as 95% confidence intervals (CI) with two-sided *P* values and risk ratios (RR). *P* values <0.05 were regarded as statistically significant. The *I*^2^ test and Cochran's *Q* test were performed to detect heterogeneity. The heterogeneity was defined as small when *I*^2^ < 50% and significant when *I*^2^ < 50%. If *I*^2^ was <50%, the fixed effect model was applied; otherwise, the random effect model was applied if *I*^2^ was >50%. We established a funnel plot to assess publication bias and determine sources of heterogeneity if more than ten studies were included to assess this endpoint.

## 3. Results

### 3.1. Features of the Studies Included and Retrieved Data

**Figure 1:**
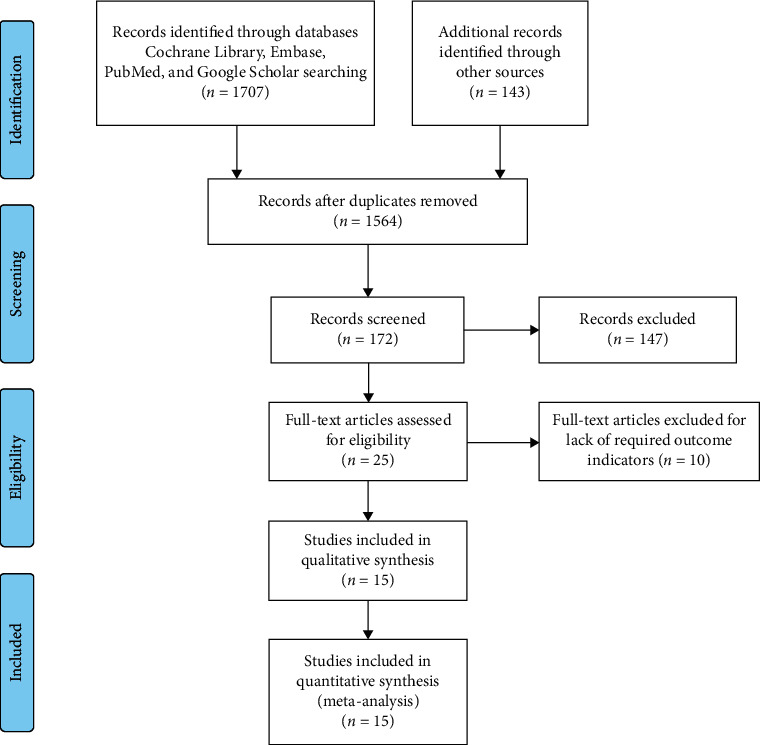
Flow diagram of the study selection process.

According to the PRISMA guidelines, we included 1564 studies which met our criteria. The abstracts and titles of these reports were scrutinized to remove ineligible studies. Additional studies were removed by screening of the full text. Finally, 15 articles [18–32] (16 trial comparisons) were enrolled which comprised 2221 patients (Figure 1). Overall, there were 1130 patients (50.9%) who received nicorandil and 1091 patients (49.1%) in the control group. Only RCTs were used in this meta-analysis. The basic features are shown in Table 1.

### 3.2. Assessment of Quality of the Studies

We applied the Cochrane Risk of Bias criteria to assess the quality of the enrolled studies and this process was done by two investigators independently. All studies were randomized controlled trials. 15 studies [18–32] provided the process of randomization. Two studies [21, 23] mentioned blinding of personnel and subjects. The quality of the literature studies is provided in Table 2.

### 3.3. Endpoints

#### 3.3.1. Periprocedural Myocardial Injury (PMI)

**Figure 2:**
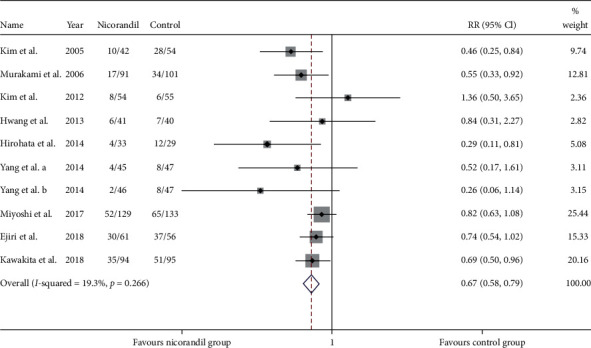
Comparison of PMI between the experimental group and the control group. RR = risk ratio; PMI = periprocedural myocardial injury.

Nine studies [18, 20–22, 24–27, 31] (10 trial comparisons) reported PMI. In total, 168 out of 636 patients in the nicorandil group experienced PMI, while 248 out of 610 patients in the control group experienced PMI. The result showed that nicorandil markedly decreased the incident of PMI compared to the control group (26.4% vs 40.7%) (RR: 0.67, 95% CI 0.58 to 0.79, *I*^2^=19.3%, heterogeneity chi-square = 11.15, *P*=0.266) (Figure 2). Thus, we applied the fixed effect model.

#### 3.3.2. Major Adverse Cardiovascular Events (MACE)

**Figure 3:**
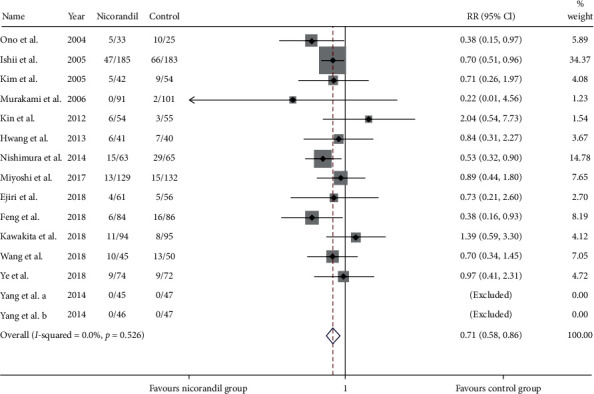
Comparison of MACE between the experimental group and the control group. RR = risk ratio; MACE = major adverse cardiovascular event.

Fourteen studies [18–32] (15 trial comparisons) reported MACE. In total, 137 out of 1097 patients in the nicorandil group experienced MACE, while 192 out of 1062 patients in the control group experienced PMI. The result showed that nicorandil markedly decreased the incident of PMI compared to the control group (12.5% vs 18.1%) (RR: 0.71, 95% CI 0.58 to 0.86, *I*^2^=0.0%, heterogeneity chi-square = 11.04, *P*=0.607) (Figure 3). Thus, we applied the fixed effect model.

#### 3.3.3. Publication Bias and Sensitivity Analysis

**Figure 4:**
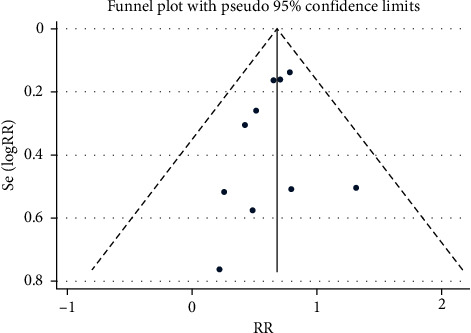
Comparison of PMI between the experimental group and the control group (funnel plot). RR = risk ratio; PMI = periprocedural myocardial injury.

**Figure 5:**
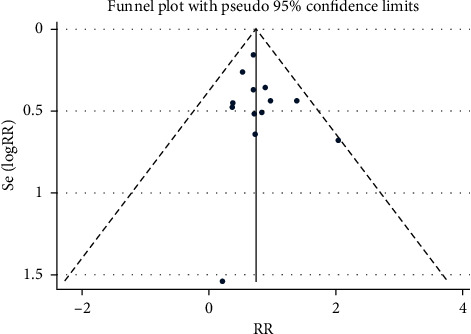
Comparison of MACE between the experimental group and the control group (funnel plot). RR = risk ratio; MACE = major adverse cardiovascular event.

**Figure 6:**
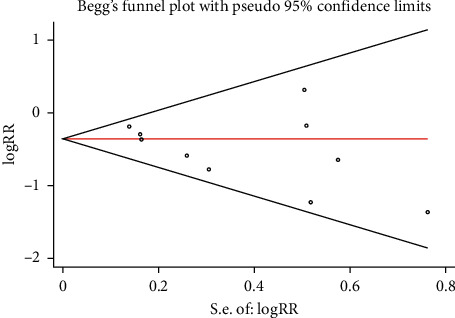
Comparison of PMI between the experimental group and the control group (Begg's test). RR = risk ratio; PMI = periprocedural myocardial injury.

**Figure 9:**
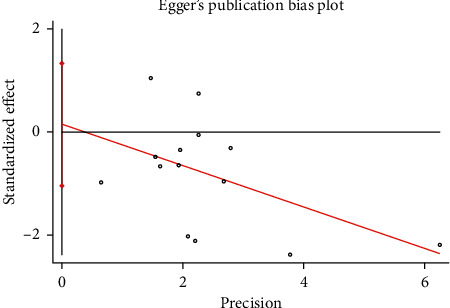
Comparison of MACE between the experimental group and the control group (Egger's test). RR = risk ratio; MACE = major adverse cardiovascular events.

**Figure 10:**
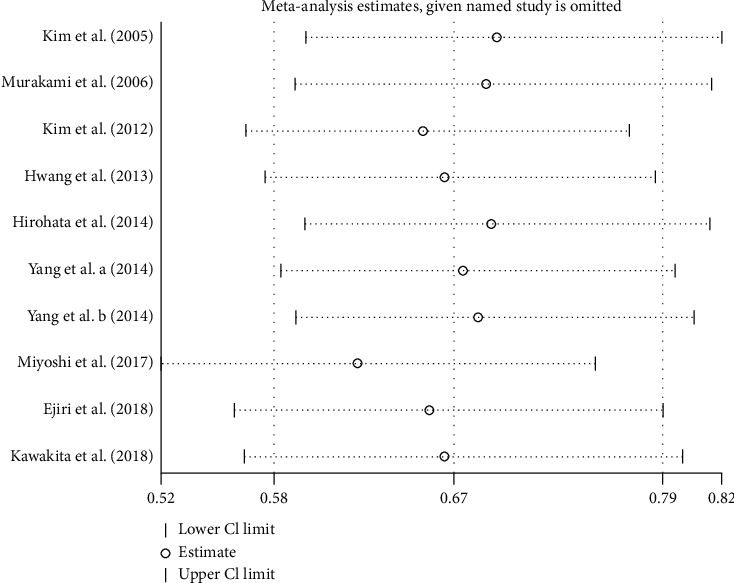
Comparison of PMI between the experimental group and the control group (sensitivity analysis). RR = risk ratio; PMI = periprocedural myocardial injury.

**Figure 11:**
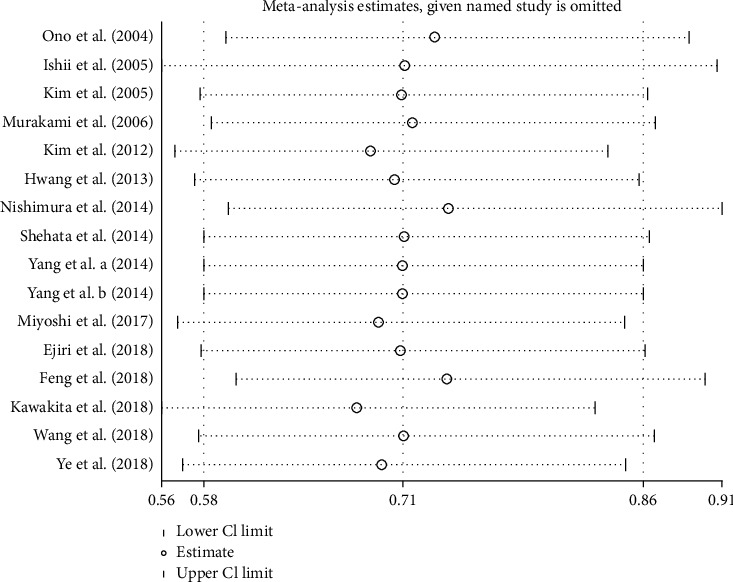
Comparison of MACE between the experimental group and the control group (sensitivity analysis). RR = risk ratio; MACE = major adverse cardiovascular events.

The funnel plot showed that there was low probability of publication bias among retrieved articles as shown in Figures 4 and 5. Begg's test and Egger's test are shown in Figures 6,9. The results of the sensitivity analysis are shown in Figures 10 and 11.

## 4. Discussion

Percutaneous coronary intervention (PCI) has become a mature technique in the treatment of coronary artery stenosis [8]. However, severe surgical complications have also been reported and perioperative myocardial injury (PMI) is the most common. Intervention with statins and glycoprotein IIb/IIIa inhibitors prior to PCI has been shown to reduce the incidence and degree of myocardial necrosis, but the incidence is still as high as 15% [33].

Nicorandil, a combined agent with an adenosine triphosphate-sensitive K (K^+-^ATP) channel agonist and nitrate preparation, could improve clinical outcomes for ischemic heart disease through relieving both microcirculation dysfunction and myocardial injury [34], but it is still uncertain whether nicorandil can effectively prevent PMI after PCI, and the preventive effect of nicorandil on myocardial injury after PCI has attracted much attention in recent years. Thus, the purpose of the systematic meta-analysis we conducted was to evaluate the myocardial protective effect of nicorandil.

Nowadays, there are only a few meta-analyses to study the relationship between the use of nicorandil before percutaneous coronary intervention (PCI) and cardiovascular events, and the conclusions are inconsistent. Li et al. [35] conducted a meta-analysis and found that nicorandil administration reduces cardiovascular events and NRP, but the study only reflected the short-term effects of combining primary PCI with nicorandil administration. The study by Zhang et al. [36] suggested that nicorandil, as an adjuvant therapy with PCI, can reduce total mortality and cardiovascular death in patients with primary PCI (PPCI) and elective PCI (EPCI) and reduce heart failure in PPCI patients. Zhu et al. [37] said that nicorandil did not reduce the overall incidence of perioperative complications and the incidence of major adverse cardiac events (MACE) in patients with angina pectoris who underwent elective PCI. Li et al. [38] suggested that nicorandil could improve clinical outcomes in terms of perioperative myocardial infarction (MI). However, the effect of nicorandil on the major adverse cerebrovascular and cardiovascular events (MACCE) risk is not obvious. Ye et al. [39] indicated that nicorandil can reduce myocardial injury and reduce the incidence of adverse reaction caused by PCI for Chinese's population but is not obvious for non-Chinese population.

Our meta-analysis evaluated the efficacy of nicorandil in the prevention of myocardial infarction and major adverse cardiovascular events (MACE) in patients after PCI. The results showed that compared with the control group, nicorandil significantly reduced the incidence of myocardial infarction and major adverse cardiovascular events (MACE) after PCI (RR: 0.67, 95% CI: 0.58 to 0.79; RR: 0.71, 95% CI: 0.58 to 0.86).

The potential clinical implications of this meta-analysis are as follows: (1) compared to previous studies, a total of 15 articles (16 trial comparisons) were included, with a larger sample size of 2,221 patients. (2) In previous studies, diabetes patients accounted for a relatively high proportion, and oral sulfonylureas were considered to inhibit the effect of nicorandil and cause bias. In this study, other diseases and drug effects were excluded, and the results were more objective. (3) Compared with previous studies, this study included literature from different regions of China, Japan, South Korea, Russia, and Egypt, and the quality of the included literature was higher.

The limitations of our meta-analysis include the following aspects: (1) the included studies are mainly from Asia, and there is a lack of randomized controlled trials from North America, Europe, and so on. (2) The mode of drug administration is not uniform. Some articles adopt the method of oral administration, some articles use the method of intravenous administration, and some articles use the method of coronary artery administration, which may lead to methodological deviation. (3) Although the articles included in our meta-analysis are randomized controlled trials, the blind methods of most studies are not clear, and there will inevitably be deviations. (4) To evaluate the myocardial protective effect of nicorandil on patients after PCI, short-term and long-term indexes need to be observed. There are few indicators in this article.

Our study suggests that we should pay attention to several aspects of randomized controlled trials in the future: (1) due to ethnic differences, it is necessary to conduct multiregional and multicenter randomized controlled trials. (2) Standardize the methods of drug use, including usage, dosage, and time of use, so as to reduce the occurrence of confusion bias as much as possible. (3) The methods of random grouping, single-blind or double-blind and their implementation are described in detail to reduce the hybrid deviation. (4) Increase the short-term and long-term indicators and increase the reliability of the results.

This meta-analysis showed that compared with the control group, nicorandil significantly reduced the incidence of myocardial infarction and major adverse cardiovascular events (MACE) after PCI, proving its reliable efficacy and safety.

## Figures and Tables

**Figure 7:**
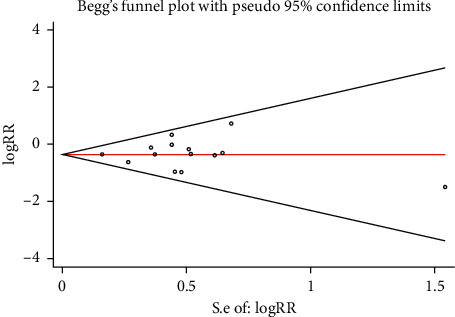
Comparison of MACE between the experimental group and the control group (Begg's test). RR = risk ratio; MACE = major adverse cardiovascular events.

**Figure 8:**
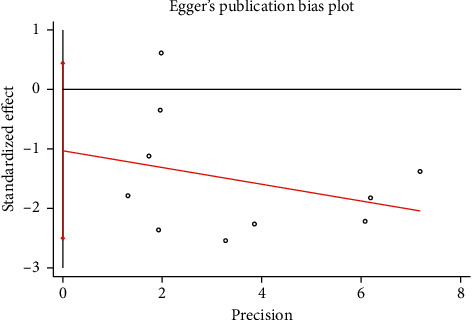
Comparison of PMI between the experimental group and the control group (Egger's test). RR = risk ratio; PMI = periprocedural myocardial injury.

**Table 1:** Characteristics of studies included in the meta-analysis.

Authors	Year	Country	Sample size		Male, no. (%)		Average age (years)	
			*E*	*C*	*E*	*C*	*E*	*C*
Ono et al. [29]	2004	Japan	33	25	22 (66.7)	16 (64.0)	64 ± 13	66 ± 12
Ishii et al. [23]	2005	Japan	185	183	144 (77.8)	154 (84.1)	63 ± 9.4	64 ± 10.1
J. Kim et al. [24]	2005	Korea	42	54	27 (64.3)	32 (59.3)	60.4 ± 11.7	61.7 ± 8.2
Murakami et al. [27]	2006	Japan	91	101	75 (82.4)	81 (80.2)	65.0 ± 9.7	66.1 ± 10.3
S. Kim et al. [25]	2012	Korea	54	55	36 (70.4)	46 (83.6)	65.5 ± 7.4	63.2 ± 9.2
Hwang et al. [22]	2013	Korea	41	40	20 (48.8)	25 (62.5)	66.2 ± 9	65.3 ± 10
Hirohata et al. [21]	2014	Japan	33	29	25 (75.8)	20 (69.0)	73 ± 13	68 ± 9
Yang et al. a [31]	2014	China	45	47	31	31	NA	NA

PMI = periprocedural myocardial injury, MACE = major adverse cardiovascular events, and PCI = percutaneous coronary intervention.

**Table 2:** Assessment of methodological quality of included studies.

Study	Bias arising from the randomisation process	Bias due to deviations from intended interventions	Bias due to missing outcome data	Bias in measurement of the outcome	Bias in selection of the reported result	Overall bias
Ono et al. [29]	Low	Low	Low	Low	Low	Low
Ishii et al. [23]	Low	Low	Low	Low	Low	Low
J. Kim et al. [24]	Low	Low	Low	Low	Low	Low
Murakami et al. [27]	Low	Low	Low	Low	Low	Low
S. Kim et al. [25]	Low	Low	Low	Low	Low	Low
Hwang et al. [22]	Low	Low	Low	Low	Low	Low
Hirohata et al. [21]	Some concerns	Low	Low	Low	Low	Some concerns
Yang et al. [31]	Low	Low	Low	Low	Low	Low
Nishimura et al. [28]	Low	Low	Low	Low	Low	Low
Miyoshi et al. [26]	Low	Low	Low	Low	Low	Low
Feng et al. [19]	Low	Low	Low	Low	Low	Low
Ye et al. [32]	Low	Low	Low	Low	Low	Low
Ejiri et al. [18]	Low	Low	Low	Low	Low	Low
Kawakita et al. [20]	Low	Low	Low	Low	Low	Low
Wang [30]	Low	Low	Low	Low	Low	Low

## Data Availability

The data used to support the findings of this study are available from the corresponding author upon request.

## Abbreviations

RCTs: Randomized controlled trials
RR: Risk ratios
CI: Confidence intervals
PMI: Periprocedural myocardial injury
MACE: Major adverse cardiovascular events
PCI: Percutaneous coronary intervention.
